# FEM Simulation and Verification of Brazing SiC Ceramic with Novel Zr-Cu Filler Metal

**DOI:** 10.3390/ma12203380

**Published:** 2019-10-16

**Authors:** Bofang Zhou, Zhichen Zeng, Yuchen Cai, Keqin Feng

**Affiliations:** 1 School of Mechanical Engineering, Sichuan University, Chengdu 610065, China; hs_zbf@163.com (B.Z.); ntcaiyuchen@126.com (Y.C.); 2 School of Materials Science and Engineering, Hubei University of Automotive Technology, Shiyan 442002, China; 3 College of Control Science and Engineering, Zhejiang University, Hangzhou 310027, China; zczeng@zju.edu.cn

**Keywords:** SiC ceramic, Zr-Cu filler metal, brazing, FEM simulation

## Abstract

Zr-Cu filler metal is proposed for SiC ceramic under special working conditions, as a novel type of the active filler metal, the difference of physical and chemical properties between SiC ceramic and Zr-Cu filler metal leads to greater residual stress in the joint, which affects the mechanical properties of brazing SiC ceramic joint. Based on the finite element method (FEM) simulation, the residual stress of the joint is simulated to guide the design of Zr-based filler metal and formulation of brazing process. The residual stress distribution of SiC ceramic joints brazed at 1200 °C with different thickness of the filler metal and cooling rate is simulated by ANSYS software. The simulation results of the residual stress are verified by brazing experiments and XRD measurements. The results show that the simulated residual stress of the joint is mainly axial compressive stress. The axial compressive stresses are the lowest when the filler metal thickness is 0.1 mm and the cooling rate is 2 °C /min, and increase with the increase of the filler metal layer thickness and cooling rate. The shear strength of the brazed SiC ceramic joint that achieves the highest with 2 °C /min is about 72 MPa, and then decreases with the increase of cooling rate. The experimental test of residual stress in different locations of the brazed SiC ceramic joint basically coincide with the FEM simulation.

## 1. Introduction

Modern ceramic materials are more and more widely used in the manufacturing industry with the rapid development of science and technology. Silicon carbide ceramic, as a kind of structural ceramics, have a series of excellent properties and been widely used in high-temperature components, such as rocket engines and space mirrors. In addition, it has potential applications in nuclear industry, space optics, and high-temperature gas filters [1,2,3]. Based on the special working conditions of SiC ceramic with the intrinsic brittleness and poor machinability, it is very difficult to fabricate parts with large and complex shape. Combining various joining methods of SiC ceramic and operating under special working conditions, our research group innovatively proposed to develop a new type of Zr-based filler metal for brazing SiC ceramic [4]. The difference of physical and chemical properties between SiC ceramic and filler metal, especially the thermal expansion coefficient and elastic modulus, leading to the larger residual stress of the brazing SiC ceramic [5,6,7]. The residual stress distribution of the SiC ceramic joint will affect its mechanical property and safety. At present, the experimental test and numerical simulation method are used for evaluating residual stress of SiC ceramic joints [8,9]. The cost of experimental test is higher, and the narrower weld seam can-not be tested. The numerical simulation method can overcome the shortcomings of the experimental test, and it is an effective method to analyze the residual stress of the joint. The finite element method simulations (FEM simulation) software, Comsol Multiphysics, is one of the lead FEM method software, includes ANSYS, MSC/NASTRAN, ABAQUS, ADINA and MARC, and ANSYS is one of the most commonly used software for welding simulation [10,11]. Therefore, this study is mainly discussed the residual stress of SiC ceramic joint is analyzed by ANSYS simulation, and verified by experimental test. However, Zr-Cu filler metal, as a novel type of filler metal, its properties are quite different from those of other filler metals or SiC ceramic, the residual stress distribution of the joint has an important influence on the design of Zr-based filler metal and formulation of joining process parameters for brazing SiC ceramic. In the paper, the residual stresses distribution of the joint between SiC ceramic and Zr-Cu filler metal under brazing conditions is investigated by ANSYS software and verified by experimental test, which provides a foundation for the design and development of Zr-based filler metal and adjusting the residual stress of joints to obtain ideal brazed joint.

## 2. FEM Simulation and Experiment Methods

### 2.1. FEM Simulation of Brazing SiC Ceramic Joint

Brazing is a transient heat transfer process which mainly involves the coupling of stress and temperature field. In order to be closer to the FEM simulation of brazing process, thermo-elastic-plastic model is adopted. A "sandwich" structure is formed for brazing SiC ceramic, which results in the non-linearity of geometry and material properties. So incremental method and virtual work principle are used to obtain the each unit stress, and then formation and distribution of the residual stress in the whole brazed joint are obtained.

The accuracy of the materials’ physical properties will directly affect the simulation results in the FEM simulation process. At the same time, 80Zr20Cu (wt.%), as a novel type of the active Zr-based filler metal (Zr-Cu filler metal), is innovatively designed for SiC ceramic under special working conditions. The main physical parameters used in FEM simulation are as follows: modulus of elasticity (E), coefficient of thermal expansion (α), yield strength (σ_s_), thermal conductivity (λ), heat capacity (c), density (ρ) and Poisson’s ratio (*ν*). The related physical properties of the SiC ceramic and Zr–Cu filler metal which were obtained by experimental measurement or calculation change with temperature are shown in Table 1. It is assumed in the FEM simulation as follow: (1) SiC ceramic only occur elastic deformation, while Zr-Cu filler metal undergo elastic and plastic deformation. (2) Convection and heat conduction between workbench and SiC ceramic are not considered in the FEM simulation process of brazing SiC ceramic, and radiation from SiC ceramic and workbench with furnace is only considered. (3) The interface of brazed joint is well bonded without defects, the physical bonding between SiC ceramic and Zr-Cu filler metal does not take into account their chemical reactions. (4) The yield strength of SiC ceramic joint is based on von Mises yield criterion when plastic deformation occurs, and equivalent plastic stress (von Mises stress) is expressed as Equation (1):

(1) σ¯={12[(σx−σy)2+(σy−σz)2+(σz−σx)2]+3(τxy2+τyz2+τzx2+)}12

In the formula, $\bar{\sigma }$ is von Mises stress; σ_x_, σ_y,_ and σ_z_ are expressed as the axial stresses in X, Y, and Z directions in turn. τ_xy_, τ_yz_, and τ_zx_ are expressed as tangential stresses.

**Figure 1:**
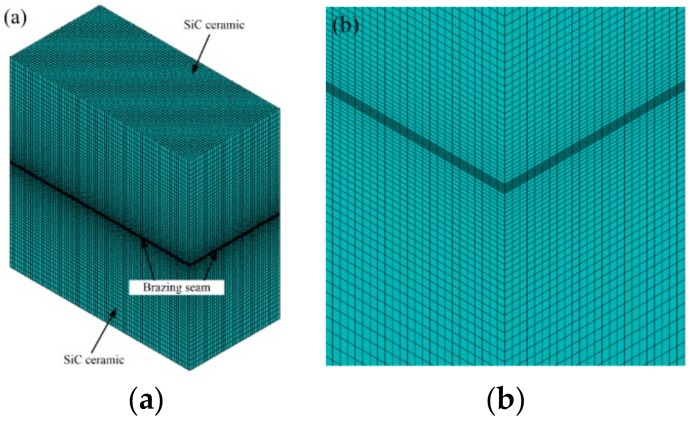
Finite element method (FEM) simulation meshing of SiC ceramic joint: (**a**) overall structure of the model, (**b**) local enlargement of the brazing seam.

The FEM simulation is built on the basis of the brazing specimen’s actual size. ANSYS software is used for FEM simulation, solid5 eight-node thermo-mechanical coupling element is selected as the mesh element type, and the non-linear algorithm chooses FULL Newton-Raphson method with fast convergence speed and automatic time step. The initial condition is room temperature at 20 °C. The brazing temperature needs to be higher than the liquidus temperature of the Zr-Cu alloy (1056 °C), so the residual stress distribution of the joints at 1200 °C is simulated [4]. The loading process of the brazed SiC ceramic is simulated to be cooled from brazing temperature to room temperature with a certain cooling rate. Relevant studies have found that the joint residual stress mainly concentrates in the nearby area of the brazing seam [5,12], and considering the accuracy of simulation calculation, the meshing of the area near the brazing seam is shown in Figure 1. This is because the non-uniform meshing can save time and reduce the computer load, while ensuring the accuracy of the FEM simulation calculation.

### 2.2. Brazing Experiment and Evaluation

**Figure 2:**
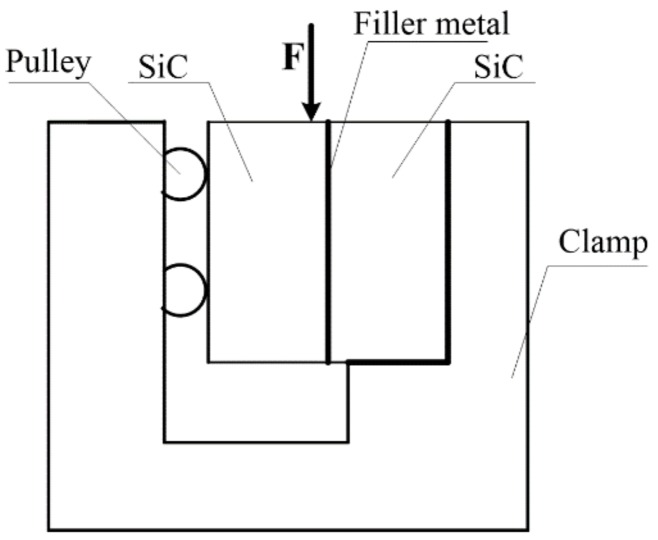
Schematic of the shear strength test.

Reactive sintered silicon carbide (RBSiC, Hua Mei fine technical ceramics, Wei fang, China) as the ceramic material was used in the brazing experiment, the related properties of the SiC ceramic in which 20 wt.% free silicon are shown in Table 2. SiC ceramic was processed in the shape of 10 × 10 × 20 mm. One side was polished with 2000^#^ diamond grinding disc and cleaned with ultrasonic wave (BK-900D, Shobakr, Jinan, China) in acetone for 30 min. Zr–Cu filler metal was processed into a certain shape, polished and cleaned by ultrasonic wave. Then a "sandwich" structure of the SiC ceramic/Zr-Cu filler metal/SiC ceramic was formed and placed in a vacuum brazing furnace (VQS-335, Darente vacuum, Shenyang, China). Under certain brazing conditions, brazing experiments were carried out to obtain brazing joints. The shear strength of brazed joints was tested by microcomputer electronic universal material testing machine (REGER-300, Reger, Shenzhen, China), and the schematic of the shear strength test is shown in Figure 2.

Residual stress of the brazing SiC ceramic joints was measured by XRD (XRD, EMPYREAN, Netherlands). The center of the brazed joint and the interface reaction layer near the SiC ceramic are used as the experimental test locations. CuZr_2_{402} is chosen as the diffraction plane when measuring residual stress in the center of the brazed joint, and its stress-free diffraction angle is 2*θ*_0_ ≈ 133.3°, while ZrC{222} is chosen as the diffraction plane when measuring the position of the interface reaction layer, and its stress-free diffraction angle is 2*θ_0_* ≈ 136.4°, the inclination angles (Ψ) are 0°, 10°, 20°, 30° and 40° respectively. In addition, the elastic modulus and Poisson’s ratio of the SiC ceramic and Zr-Cu filler metal are shown in Table 1. The formula for calculating residual stress is as Equation (2).

(2) $$\sigma_{\emptyset }=-\frac{E}{2\left(1+\nu \right)}\cos\theta_{0}\frac{\pi }{180^{{}^{\circ}}}\frac{\partial \left(2\sigma_{\emptyset ,\Psi }\right)}{\partial \left(\sin^{2}\Psi \right)}$$

In the formula, *σ_ϕ_* is the residual stress value of a part of the brazed joint (MPa), *E* is the elastic modulus of the test location, *ν* is the Poisson’s ratio of the material, *θ*_0_ is the Bragg angle of the diffraction peak in the non-stress state, *Ψ* is the given measuring angle for measurement, *θ_ϕ,Ψ_* is the Bragg angle of the diffraction peak under stress condition.

## 3. Results and Discussion

### 3.1. Von Mises Stress of the FEM Simulation Joint

**Figure 3:**
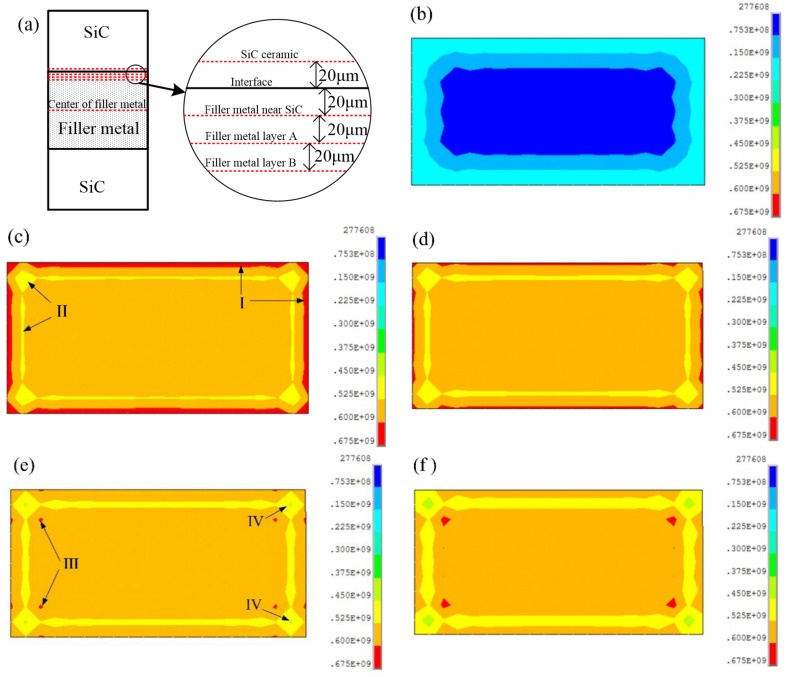
Von Mises stress of the different regions of the SiC ceramic joints at 1200 °C: (**a**) schematic diagram of brazing joint slice, (**b**) SiC ceramic side, (**c**) filler metal near SiC ceramic, (**d**) filler metal layer A, (**e**) filler metal layer B, (**f**) center of the filler metal.

Figure 3 shows that the von Mises stress in different internal regions of the FEM simulation joint by slicing method at 1200 °C. As shown in Figure 3a, the slice diagram includes the SiC ceramic side of the joint, the filler metal layer near the SiC ceramic, the filler metal layer A, the filler metal layer B and the center of the filler metal layer, with a spacing about 20 μm. The von Mises stress in the central region of SiC ceramic is the smallest from Figure 3b, and the von Mises stress gradually increases from the central region to the edge. However, the von Mises stress of the filler metal layer is relatively large and distributed in a certain region near the edge. The maximum von Mises stress occurs mainly at the joint between SiC ceramic and filler metal, and the stress in region I gradually decreases from the filler metal layer near SiC ceramics to the center of the filler metal layer, and the von Mises stress decreases gradually from region I to region II. A similar wide enclosed area is formed in region II, which can realize the effective bonding between filler metal and SiC ceramic [13]. The main reason is that the residual stress in region II is relatively smaller, which shows that the material performance matches. From the filler metal near SiC ceramic to the center of the filler metal layer, the regions (II, III, IV) become larger, moreover, the smaller von Mises stress in IV region is beneficial to reduce the residual stress of the joint, while von Mises stress of the III region is opposite. Therefore, it can be inferred that the most disadvantageous part of the joint is the filler metal layer near the SiC ceramic. At the same time, the von Mises stress distribution of Zr-Cu alloy is similar to that of related alloys and SiC ceramic [14,15], but the properties of Zr-Cu alloy are closer to that of SiC ceramic, resulting in relatively small residual stress, which highlights the innovation and importance of Zr-Cu filler metal design. In order to obtain an ideal SiC ceramic brazing joint, it is necessary to design Zr-based filler metal and optimize brazing process parameters so as to adjust the von Mises stress distribution of the joint.

According to the von Mises stress distribution of the SiC ceramic joint, combined with von Mises criterion and the fluidity and restriction conditions of the filler metal in actual brazing process, the main residual stress of the joint is the axial stress (σ_y_), and the stress concentration mainly distributes at the four edges of the brazing joint. So the stress distribution along the edges of brazed joints under different thickness of the filler metal and cooling rates will be mainly investigated.

### 3.2. The Influence of Filler Metal Thickness on Axial Stress of Joint

**Figure 4:**
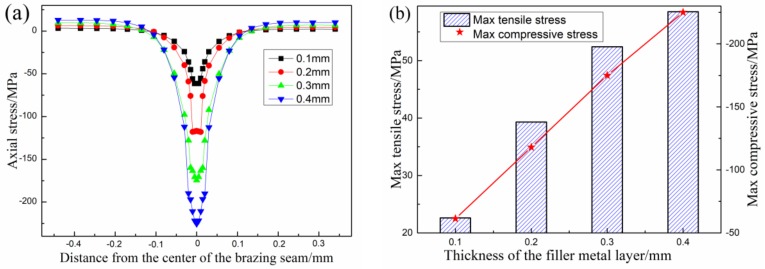
Axis stress (σ_y_) distribution of the SiC ceramic joints with different thickness of filler metal: (**a**) distribution on the edge of joint, (**b**) Maximum tensile and compressive stresses.

The simulated axial stress distributions of the joints with different filler metal thickness are shown in Figure 4. It can be found that the distribution of axial stress is mainly symmetrical in the center of the brazing seam, and the maximum axial stress that occurs in the filler metal layer is compressive stress, the compressive stress decreases gradually with the transition of the brazing seam center to SiC ceramic, and the stress changes at the interface of the joint, and then the tensile stress appears in the SiC ceramic finally. The compressive stress at the edge of the joint with the same thickness of filler metal layer is much greater than the tensile stress, which indicates that the axial stress of the joint is mainly compressive stress. This is mainly due to the difference of thermal expansion coefficient (α) and elastic modulus (E) between SiC ceramic and Zr–Cu filler metal at high temperature, which indicates that Zr-based filler metal should be optimized to obtain brazed SiC ceramic joints with lower stress. It is found that the maximum tensile stress or compressive stress of the joints increases with the increase of the filler metal thickness from the Figure 4b. In conclusion, it is necessary to reduce the residual stress, especially the compressive stress, at the same time, control the thickness of the filler metal layer, in order to gain the joints with excellent mechanical properties.

### 3.3. The Effect of Different Cooling Rates on the Axial Stress of Joint

**Figure 5:**
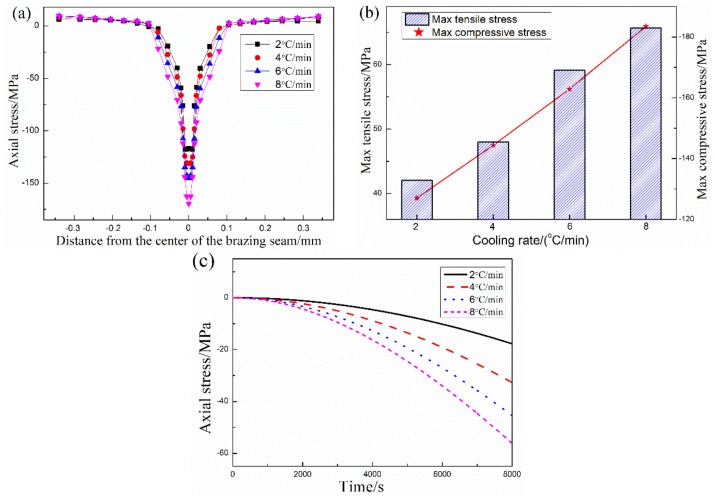
The axial stress (σ_y_) distribution of SiC ceramic joints with different cooling rates: (**a**) the edge distribution, (**b**) Maximum tensile and compressive stresses, (**c**) distribution of the node A.

According to the stress distribution of the SiC ceramic joint with different thickness of filler metal, and preparation conditions of the filler metal, the thickness of filler metal layer is 0.2 mm for further study. The axial residual stress of joints that brazing a temperature at 1200 °C with different cooling rates are shown in Figure 5. It can be seen that the axial stress of the joints, presenting compressive stress, mainly concentrates on the filler metal layer at the same cooling rate. The compressive stress of the joints gradually decreases from the center of brazing seam to the SiC ceramic, and stress of the area near the SiC ceramic is transformed into tensile stress, and finally stabilizes to a certain value. As shown in Figure 5b, the maximum compressive stresses of the joint increases with the increase of cooling rate, and is higher than its maximum tensile stress under the same condition. Meanwhile, the change of the axial stress at a point A in the center of the brazing seam with time under different cooling rates is investigated. The axial compressive stress increases with the increase of cooling time from Figure 5c. As a result, the axial stress of the joint enhances with the increase of cooling rate due to the stress of the joint is not being released under rapid cooling.

### 3.4. Verification of FEM Simulation 

**Figure 6:**
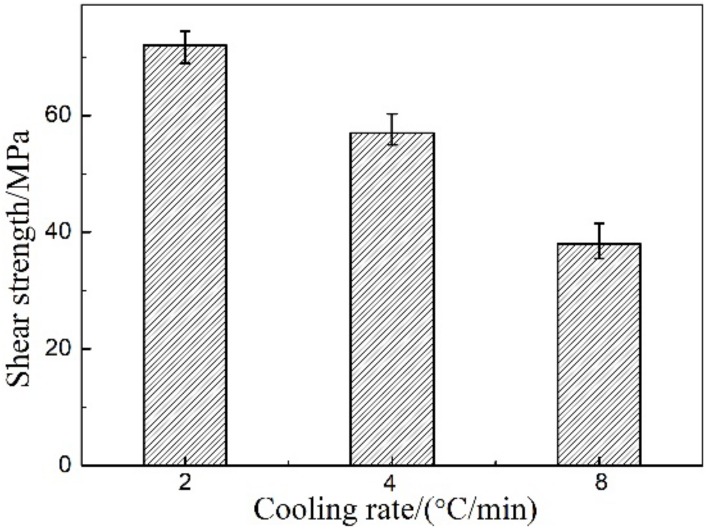
Shear strength of the brazed SiC ceramic joint with different cooling rate.

According to the results of FEM simulation and considering the experimental conditions, SiC ceramic and Zr-Cu filler metal with 0.2 mm thickness were heated to brazing temperature 1200 °C, and holding for 20 min, and then cooled to room temperature with the different cooling rate. It can be found that brazed joints can achieve chemical metallurgical bonding between SiC ceramic and Zr-Cu filler metal. The shear strength of the brazed SiC ceramic joints with different cooling rates is shown in Figure 6. It can be seen that the shear strength of the joints that achieves the highest with 2 °C/min is about 72 MPa, and then decreases with the increase of cooling rate. At the same time, it was found that the shear fracture location of the joint was mainly in the interfacial reaction layer, and there were a few SiC ceramic particles in the fracture. The main reason is that the physical properties of SiC ceramic and filler metal are quite different, especially their thermal expansion coefficient and elastic modulus. The stress of SiC ceramic is mainly tensile stress and that of the filler metal layer is compressive stress, and the axial stress mainly concentrates on the center of the filler metal layer. The faster the cooling rate is, the greater the axial stress concentration of the filler metal is, which will lead to the decrease of the shear strength of the brazing SiC ceramic joints.

**Figure 7:**
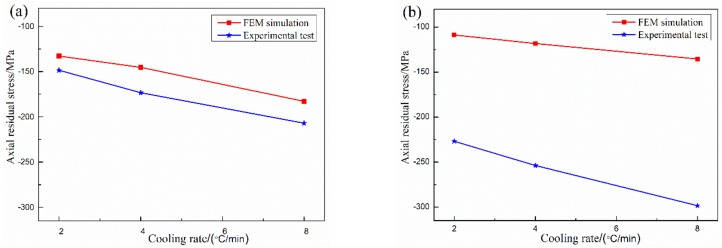
Residual stresses comparisons between the experimental test and the FEM simulation with different cooling rate: (**a**) the center area of joint, (**b**) the interface reaction layer near SiC ceramic.

The residual stresses of the experimental brazing joints with different cooling rates are compared with the FEM simulation as shown in Figure 7. The residual stresses in the central region of the joint and the area near the interface of SiC ceramic are all compressive stresses. The compressive stresses of the joints increase with the increase of cooling rate. The variation of residual compressive stresses in the central region of the joint is consistent with that in the experimental measurement. However, the residual compressive stresses in the central region of the joint are slightly larger than those in the FEM simulation at the same cooling rate. In the area near the interface of SiC ceramic, the joining of the filler metal and SiC ceramic is assumed to be a physical bonding in the FEM simulation process, and the interface reaction between Zr-Cu filler metal and SiC ceramic is not considered, resulting in the residual compressive stress of the reaction layer is much larger than that of the FEM simulation at the same cooling rate. If the results of FEM simulation are closer to the experimental test, the interface reaction layer should be introduced into the FEM simulation process [4], the current detection methods are difficult to obtain the interfacial reaction layer material relevant physical performance, especially the properties of the reaction layer material change with temperature. Only a few references are involved [16,17], thus the physical properties of the interface reaction layer material need to be further investigated. Although there are some errors between the experimental test and FEM simulation of brazed SiC ceramic joints, the stress changing trend of the experimental test can basically better verify the FEM simulation between SiC ceramic and Zr-Cu filler metal.

## 4. Conclusions

(1) Axial stress is an important component of residual stress in SiC ceramic brazing joint, and the stress concentration mainly distributes symmetrically along the four edges of the SiC ceramic joint. Axial stress is mainly composed of compressive stress and tensile stress, the compressive stress decreases gradually from the center of the brazing seam to SiC ceramic, and transforms into tensile stress at the side of SiC ceramic. 

(2) The maximum compressive stress occurs in the center of the brazing seam, while the maximum tensile stress occurs on the SiC ceramic side. The maximum compressive stress of the SiC ceramic joint is much greater than its tensile stress, which results in the joint presenting compressive stress. The compressive stresses are the lowest when the filler metal thickness is 0.1 mm and the cooling rate is 2 °C/min, and increase with the increase of the filler metal layer thickness and cooling rate. 

(3) The shear strength of the brazed SiC ceramic joint is the highest (i.e., 72 MPa) with brazed at 1200 °C and 2 °C/min of the cooling rate, and then decreases with the increase of cooling rate. The experimental residual stress is consistent with the results of FEM simulation, so the residual stress of the brazed SiC ceramic joint using experimental test can basically better verify the stress of the FEM simulation. 

## Figures and Tables

**Table 1:** Physical properties of SiC ceramic and filler metal (80Zr20Cu (wt.%)).

Materials	T (K)	E (GPa)	A (10^−6^/K)	σ_s_ (MPa)	Λ (W/m/K)	c (J/kgK)	Ρ (kg/m^3^)	*ν*
SiC	293	330	4.5	-	28.6	670	3120	0.19
	473	326	4.8	-	32.8	813	3120	0.19
	673	318	5.2	-	37.1	1088	3120	0.19
	873	309	5.5	-	41.7	1173	3120	0.19
	973	302	5.6	-	43.4	1202	3120	0.19
	1073	291	5.9	-	45.2	1227	3120	0.19
Filler metal	293	117	7.4	398	329	280	6690	0.33
	473	114	7.5	382	391	426	6690	0.33
	673	105	7.7	363	45.8	542	6690	0.33
	873	99	8.1	304	52.6	667	6690	0.33
	973	92	8.7	236	58.7	734	6690	0.33
	1073	76	12.9	128	65.4	792	6690	0.33

**Table 2:** Properties of reactive sintered silicon carbide (RBSiC) ceramic.

Properties	Maximum Temperature of Application	Density	Open Porosity	Bending Strength	Modulus of Elasticity	Thermal Conductivity	Coefficient of Thermal Expansion
RBSiC	1380 °C	>3.02 g/cm^3^	<0.1%	280 MPa	300 GPa	74 W/mK	4.5 × 10^−6^ K^−1^
